# Rare but There: Ceftriaxone-Induced Neutropenia in a Patient With a Brain Abscess

**DOI:** 10.7759/cureus.63411

**Published:** 2024-06-28

**Authors:** Muhammad Naseem, Maheen, Safi Ullah, Qaidar Alizai, Ali Motie

**Affiliations:** 1 Neuro-Rehab, Somerset NHS Foundation Trust, Taunton, GBR; 2 General Medicine, eSHIFA, Shifa Integrated Healthcare Technologies (Private) Limited, Islamabad, PAK; 3 Emergency Medicine, University Hospital Galway, Galway, IRL; 4 Surgery, Hayatabad Medical Complex Peshawar, Peshawar, PAK

**Keywords:** cerebral abscess, gcsf role in treatment of neutropenia, reversible neutropenia, drug induced neutropenia, ceftriaxone-induced neutropenia

## Abstract

This case report details the case of a 70-year-old man with Marfan syndrome and hypertension who developed neutropenia after an eight-week course of ceftriaxone, used to treat a brain abscess. Initially presenting with tonic-clonic seizures and headaches, his condition was managed with ceftriaxone and metronidazole. The subsequent drop in neutrophil counts from 7.54 × 10^9/L to 0.87 × 10^9/L leads to the discontinuation of ceftriaxone and the administration of granulocyte-colony stimulating factor (G-CSF), which effectively restored the neutrophil levels. This case highlights that clinicians should be aware of ceftriaxone-induced neutropenia as a potential complication, especially in patients undergoing prolonged therapy. Regular monitoring and timely management are essential for patient safety and favorable outcomes.

## Introduction

Ceftriaxone, a widely used beta-lactam antibiotic, has been associated with the rare but significant adverse effect of inducing neutropenia. Although the overall incidence of non-chemotherapy idiosyncratic drug-induced neutropenia ranges between 2.4 and 15.4 cases per million annually [1], beta-lactams like ceftriaxone are frequently implicated [2,3]. The mechanism underlying this condition involves both immune-mediated destruction and direct toxicity affecting neutrophil production, though the exact pathways remain incompletely understood [4]. Therefore, the clinical community must be aware of this potential complication, as early recognition and intervention can significantly alter patient outcomes. The importance of this awareness is underscored by this case of a patient diagnosed with a brain abscess, where ceftriaxone-induced neutropenia gave rise to additional challenges in management and required a personalized therapeutic approach. Studies have suggested that monitoring strategies and risk assessments be more routinely integrated into clinical practice when initiating treatment with high-risk drugs such as ceftriaxone [5].

## Case presentation

A 70-year-old male patient with a background of Marfan syndrome and hypertension presented to the emergency department with a history of three tonic-clonic seizures at home. He reported a several-day history of headaches, limb weakness, and decreased mobility. On examination, the lethargic and septic-looking patient was lying on the bed with a fever (temperature 102 F), tachycardia (heart rate: 105 bpm), tachypnea (respiratory rate: 23 bpm), and decreased power equally in both upper and lower limbs.

**Figure 1:**
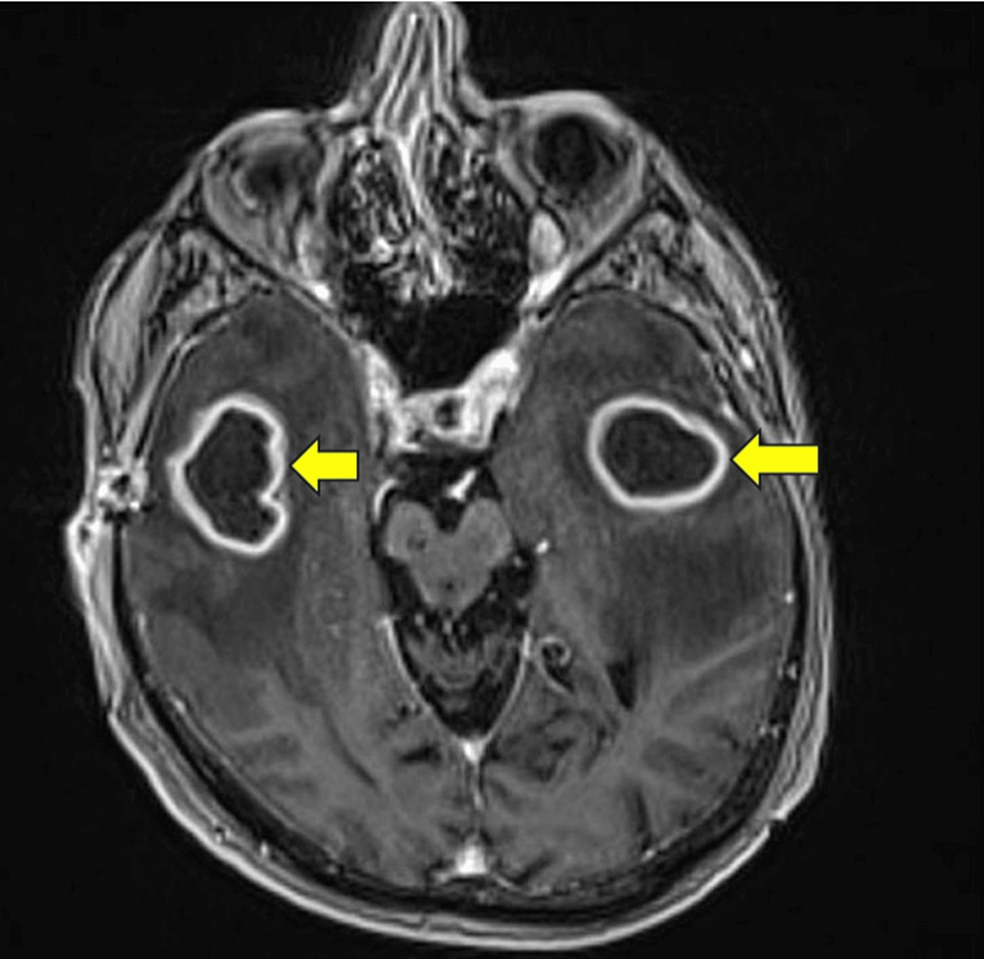
Cross-sectional view Contrast-enhanced magnetic resonance imaging of the head shows ring-enhancing lesions (yellow arrows) in bilateral temporal lobes with a central hypointense area (cystic) and a thick rim, along with surrounding regional edema of the temporal lobes.

**Figure 2:**
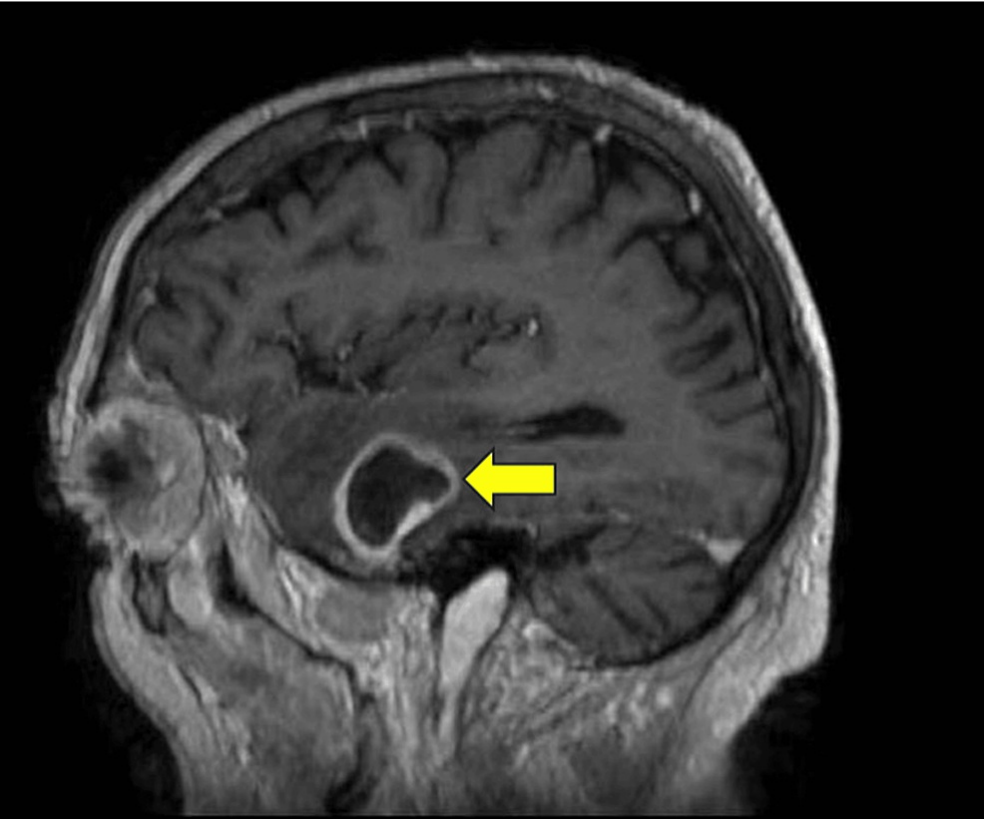
Sagittal plane Contrast-enhanced magnetic resonance imaging of the head shows a ring-enhancing lesion (yellow arrow) in the left temporal lobe with a central hypointense area (cystic) and a thick rim, along with surrounding regional edema of the temporal lobe.

His initial blood workup revealed neutrophilic leukocytosis (neutrophils: 7.54 × 10^9/L), and magnetic resonance imaging (MRI) of his brain revealed three ring-enhancing lesions (left side 30 mm; right side 31 mm) within the supratentorial brain parenchyma along with vasogenic edema in the bilateral temporal lobes suggestive of cerebral abscess (Figures 1, 2). His blood culture was positive for Streptococcus mitis/oralis, the source of which was a dental abscess, for which he underwent dental extraction. The rest of the workup, including thoracoabdominopelvic (TAP), computed tomography (CT), and echocardiography, was unremarkable.

The patient was admitted under the care of neurosurgery, and after providing informed consent, he underwent craniectomy with drainage of the intracranial abscesses. The patient was started on ceftriaxone 2 gm IV twice a day (BD) and metronidazole 500 mg IV three times a day (TDS). On a follow-up MRI of the head eight weeks later, we observed a significant reduction in the size of the temporal lobe abscesses bilaterally. Antibiotics were continued for the persistent disease. However, after eight weeks, the patient's neutrophil and WBC counts decreased below the normal range (Table 1).

**Table 1:** White blood cell and neutrophil counts during the disease course This table summarizes the fluctuations in white blood cell and neutrophil counts at various stages of the patient's treatment and recovery. The reference range of WBC is 4.0-11.0 ×10^9/L, and that of neutrophils is 2.0-7.5 ×10^9/L. Bold numbers indicate abnormally low values, reflecting instances of neutropenia. G-CSF: granulocyte-colony stimulating factor

Timeline	WBC (×10^9/L)	Neutrophils (×10^9/L)
On admission	11.03	7.54
After 8 weeks	3.09	1.42
After stopping ceftriaxone & G-CSF	21.06	16.80
10 days post-ceftriaxone discontinuation	2.55	0.93
After second G-CSF course	28.73	25.73
2 weeks post-discharge	4.14	2.64

After discussion with the microbiology team, ceftriaxone was stopped on suspicion of ceftriaxone-induced neutropenia, and the patient was started on meropenem 1 gm three times a day. We also administered two courses of granulocyte-colony stimulating factor (G-CSF) 300 µg once a day subcutaneously for three days with substantial improvement, which substantially improved the neutrophil count (Table 1).

However, his neutrophil count dropped again after 10 days of stopping ceftriaxone treatment. G-CSF was given again, which boosted his WBC and neutrophil count.

Another MRI performed 13 weeks after admission showed a significant reduction in his abscesses (on the left side, reduced to 17 mm from 20 mm; on the right side, reduced to 13 mm from 22 mm), and the patient was discharged home without antibiotics. Repeated blood tests with his general practitioner two weeks after discharge revealed a WBC of 4.14 × 10^9/L and a neutrophil count of 2.64 × 10^9/L (Table 1).

## Discussion

This case highlights the critical need to recognize ceftriaxone-induced neutropenia as a serious but rare complication of prolonged antibiotic therapy. The pathophysiology of antibiotic-induced neutropenia is not well understood but is thought to be multifactorial, involving both immunologic mechanisms and direct myelosuppressive effects [6].

Our patient developed neutropenia after eight weeks of therapy with ceftriaxone. This timeline is consistent with findings from Cimino et al., who observed onset times ranging from eight to 37 days, demonstrating the variability and unpredictability of this adverse effect [7]. Such variability underscores the necessity for continuous monitoring of blood counts throughout antibiotic therapy to detect and manage potential adverse effects timely.

Upon the development of neutropenia, the primary intervention involved discontinuing ceftriaxone and administering granulocyte-colony stimulating factor (G-CSF), which facilitated a rapid recovery of neutrophil counts. This management approach aligns with existing literature, which suggests that the immediate withdrawal of the offending drug is vital and that G-CSF should be considered for managing prolonged cases of drug-induced neutropenia [8,9]. These recommendations are supported by studies like those of Andersohn et al., which have categorized various drugs according to their potential to induce neutropenia and emphasized the effectiveness of prompt treatment interventions [6].

Furthermore, the therapeutic approach involved the administration of intravenous meropenem, a broad-spectrum antibiotic, highlighting its vital role in managing bacterial infections in severe cases such as brain abscesses [10]. Also, this case highlights the importance of interdisciplinary collaboration in managing complex cases like this, where multiple specialties contribute to the diagnosis, management, and monitoring of the patient. Such collaboration is essential for preventing complications and achieving favorable outcomes. 

Although this report contributes to the body of literature on ceftriaxone-induced neutropenia, it also underlines the gaps in our understanding of its pathophysiology. More research is needed to explain the mechanisms by which ceftriaxone and similar drugs induce neutropenia. Despite its limitations, this case underscores an important drug complication that will help with the timely identification of such cases in the future. Future studies could provide insights that lead to improved prevention and management strategies for this and similar drug-related complications.

## Conclusions

Ceftriaxone-induced neutropenia, though rare, is a significant clinical challenge, underlining the necessity for heightened awareness among physicians. This case study exemplifies the imperative need for careful monitoring of blood counts in patients undergoing prolonged antibiotic therapy. The prompt recognition and management of neutropenia is essential; discontinuing the offending agent and administering granulocyte-colony stimulating factor (G-CSF) can timely reverse the condition, thereby preventing severe outcomes. Moreover, interdisciplinary collaboration is essential in managing complex clinical cases, and it also serves as a reminder of the dynamic nature of patient care, where ongoing education and awareness are vital in improving patient outcomes.

## References

[1] Curtis BR (2017). Non-chemotherapy drug-induced neutropenia: key points to manage the challenges. Hematology Am Soc Hematol Educ Program.

[2] Satake K, Iijima K (2023). Ceftriaxone-induced neutropenia successfully treated with alternative β-lactam antibiotics: a case report and review of the literature. Cureus.

[3] Duncan CJ, Evans TJ, Seaton RA (2010). Ceftriaxone-related agranulocytosis during outpatient parenteral antibiotic therapy. Journal of Antimicrobial Chemotherapy.

[4] Mistry R, Rawson TM, Troise O, Mughal N, Moore LS, Hughes S (2022). Haematological and hepatic adverse effects of ceftriaxone in ambulatory care: a dual-centre retrospective observational analysis of standard vs high dose. BMC Infect Dis.

[5] Joszt L (2017). Diagnosing non-chemotherapy drug-induced neutropenia. AJMC.

[6] Andersohn F, Konzen C, Garbe E (2007). Systematic review: agranulocytosis induced by nonchemotherapy drugs. Ann Intern Med.

[7] Cimino M A, Rotstein C, Slaughter RL, Emrich LJ (1987). Relationship of serum antibiotic concentrations to nephrotoxicity in cancer patients receiving concurrent aminoglycoside and vancomycin therapy. Am J Med.

[8] Gibson C, Berliner N (2014). How we evaluate and treat neutropenia in adults. Blood.

[9] Tamura K, Stecher G, Peterson D, Filipski A, Kumar S (2013). MEGA6: Molecular Evolutionary Genetics Analysis version 6.0. Mol Biol Evol.

[10] Bečulić H, Begagić E, Skomorac R, Jusić A, Selimović E, Čejvan L, Pojskić M (2023). Brain abscess secondary to an apparently benign transorbital injury: an infrequent case report with literature review. Anatomia.

